# Sociodemographic factors associated with anemia among reproductive age women in Mali; evidenced by Mali malaria indicator survey 2021: spatial and multilevel mixed effect model analysis

**DOI:** 10.1186/s12905-023-02351-x

**Published:** 2023-05-27

**Authors:** Gosa Mankelkl, Beletu Kinfe

**Affiliations:** 1 Present Address: Department of Biomedical, College of Medicine and Health Science, Wollo University, Dessie, Ethiopia; 2 College of Medicine and Health Science, Wollo University, Dessie, Ethiopia

**Keywords:** Sociodemographic factors, Anemia, Multilevel, Spatial, Mali

## Abstract

**Introduction:**

Anemia is a severe global public health problem that threatens human health as well as social and economic development in both developing and developed nations. Anemia is a significant public health issue because; it affects people from all backgrounds. Anemia affected about one-third of non-pregnant women, 41.8% of pregnant women, and more than a quarter of the world’s population. Any stage of a woman’s life might result in anemia, due to physiological factors, infections, hormonal imbalances, pregnancy related complications, genetic factors, nutritional deficiency and environmental factors. Mali is a developing country with substantial anemia prevalence, particularly in the developing areas. In order to reduce anemia among women of reproductive age, the Mali government worked to enhance preventative and integrative interventions. One of the government’s objectives is to reduce the prevalence of anemia in order to decrease maternal and infant mortality and morbidity.

**Methods:**

Secondary data analysis was conducted using data from Mali Malaria Indicator Survey 2021 datasets. The study comprised a total of 10,765 reproductive-age women. Spatial and multilevel mixed effect analysis, chi-square, bivariate and multivariate logistic regression were employed on determinant factors of anemia among reproductive age women in Mali. Finally, the percentage and odd ratio, its 95% confidence intervals, and the result of spatial analysis were reported.

**Results:**

This study includes a total weighted sample of 10,765 reproductive-age women from Mali Malaria Indicator Survey 2021. The prevalence of anemia was 38%. Of them, 1.4%, were severely anemic, while 23.5% and 13.1% were moderately and mildly anemic, respectively in Mali. In the spatial analysis, the spatial distribution of anemia showed that a higher proportion of anemia found in southern and south west region of Mali. The northern and north east region of Mali had a low of proportion of anemia. being youngest age [20–24] years [ AOR = 0.817; 95% CI = (0.638,1.047); P = 0.000], attending higher education [AOR = 0.401; 95% CI= (0.278,0.579); P = 0.000], being male headed household [AOR = 0.653; 95% CI= (0.536,0.794); P = 0.000] and being richest [AOR = 0.629; 95% CI= (0.524,0.754) P = 0.000] were protective factors for anemia among reproductive age women. In contrast to this, living in rural area [ AOR = 1.053; 95% CI = (0.880,1.260); P = 0.000], being animist religion follower [AOR = 3.10; 95% CI= (0.763,12.623) P = 0.04], using unimproved drinking water sources [AOR = 1.117; CI= (1.017,1.228); P = 0.021} and using unimproved toilet facility [AOR = 1.018; CI= (0.917,1.130); P = 0.041} were considered as the risk factors for anemia among reproductive age women.

**Conclusion:**

In this study, anemia was linked to socio-demographic characteristics, and there were regional variations in the frequency of anemia among women of reproductive age. The most important measures to prevent anemia among women of reproductive age in Mali included empowering women to have higher levels of education, raising the wealth index, rise in awareness of improved drinking water sources and toilet facilities, spreading anemia education through religiously acceptable routes, and using an integrated approach to prevention and intervention in high-prevalent regions of the country.

## Introduction

Anemia is characterized by a low red blood cell count or hemoglobin concentration per unit volume with in peripheral blood [1]. When Hb concentration falls between 8 and 10.9 g/dL, anemia in nonpregnant women (NPW) is termed severe, moderate when it falls between 11 and 11.9 g/dL, and mild when it falls between 11 and 11.9 g/dL [2]. Anemia is a serious worldwide public health issue that affects both developing and developed countries, causing a threat to human health, social and economic development [3]. Anemia is considered a public health issue or problem by the WHO if population surveys reveal anemia prevalence of 5.0% or greater [4]. Anemia is a serious public health issue in low-income nations because it affects people from all walks of life. Anemia affects more than one-third of persons in the Middle East, the majority of whom are women, due to iron deficiency (ID) or a combination of other factors [5].

Anemia is one of the most prevalent nutritional deficiency illnesses worldwide, affecting more than a quarter of the global population. Pregnant women (41.8%) and almost one-third of non-pregnant women (30.2%) are both anemic [6]. Women are anemic at any stage of their lives: whether it be from menstrual bleeding disorders, pre-pregnancy iron deficiency, pregnancy anemia, postpartum anemia, stress and depression in the premenopausal stage, or anemia caused by hormonal imbalance in the postmenopausal stage [7]. In addition to this Anemia can be caused by a variety of factors, including iron deficiency, infections, genetics, and other dietary deficits [8].

About half of anemia cases are thought to be caused by iron deficiency, but the proportion likely fluctuates among demographic groups and in various places, depending on the local environment [9]. Because of their increased need for iron during pregnancy, breastfeeding, menstrual blood loss, and dietary inadequacies during their reproductive cycle, iron insufficiency is frequent among women of reproductive age [10].

Anemia is a multifactorial disease that can act both as a risk factor or a consequence of diseases which may affect nervous system, respiratory and circulatory system, skin mucous membrane, digestive system, endocrine system [11]. Maternal anemia is associated with maternal and child morbidity and mortality such, as increased risk of miscarriage, stillbirth, prematurity and low birth weight of the baby [12]. Anemia in women of reproductive age is a serious public health issue in developing nations, with long-term consequences for women’s health, their children’s health, and society’s economic prosperity [10]. The 65th World Health Assembly, held in 2012, endorsed an action plan and global objectives for maternal, newborn, and child nutrition, including a goal to reduce anemia prevalence in women of reproductive age by half by 2025, compared to 2011 levels [13].

In developing countries, anemia is a major public health concern for women of reproductive age [14]. There have been studies in Mali on the overall prevalence of anemia among reproductive-age women, but less emphasis has been dedicated to the spatial variation of anemia reproductive women. In Mali due to the lack of updated and reliable figures on spatial pattern of anemia, it is difficult to establish policies and programs for the prevention of anemia and to take an intervention to decrease anemia prevalence across the region. Therefore, the major objective of this study was to evaluate spatial variations in anemia and the contributing factors of anemia among women of reproductive age in Mali. It is expected to have the following significance: First, it is important for the stakeholders to understand various factors that contribute to anemia. Secondly, the findings of this study would provide better evidence for policymakers, ministry of health and other stakeholders, which in turn might enable designing and executing appropriate interventions at different levels to decrease anemia among reproductive age women.

## Methods and data source

### Study design, setting and period

The secondary data for this analysis were obtained from 2021 Mali Malaria Indicator Survey that was found at DHS portal of (https://dhsprogram.com/data/dataset_admin/index.cfm). The sample for the 2021 Mali Malaria Indicator Survey was sorted and chosen in two steps. The stratification of each region into urban and rural areas produced 17 sampling strata. In each stratum, SE samples were individually chosen in two steps. Each stratum’s sample was randomly selected with a certain allocation. Using a systematic drawing with probability proportionate to size, 261 sections of enumerations (SE) were selected at the first level from the list of SEs created during the General Census of Population and Housing (RGPH) conducted in 2009. The size of the ES is the number of households. If an ES is very vast (more than 300 households) and just a portion of it has been chosen, a cluster corresponds to an ES or a portion of an ES for this procedure. Following the creation of the primary units or clusters, each of the clusters drawn between July and August 2021 had its households counted and its map updated. It was able to offer a list of households from which a second-degree draw had been made due to this operation. Before counting the households, ES with more than 300 were separated into segments, only one of which was included in the sample. The lists of households that were generated served as a sampling frame for choosing the households in the subsequent step. A sample of 26 households per cluster with systematic drawing and equal probability was drawn at the second stage of the drawing. 6,786 households in all were chosen, 1,794 urbans in 69 clusters and 4,992 rural in 192 clusters. The Woman’s Questionnaire was used to conduct interviews with all women aged 15 to 49 who were either visitors or long-term residents of the selected households who were there the night before the study. 10,765 women had interviews conducted with them. 8103 of the 10,765 women who were interviewed were from a rural area, while 2662 were from an urban area [15]. Since the outcome variable for this study was anemia status among reproductive age women .so, the final sample size for this analysis was 10,765.

### Study variables

#### The outcome variable

for this study was the anemia status, which was coded as “0” if the women was anemic (mild, moderate and severe anemia) and “1” if the women was not anemic.

#### Individual-level variable

maternal age, educational status, religion, sex of household, wealth index, source of drinking water and types of toilet facility.

#### Community-level variable

Region and place of residence.

### Data management and analysis

In all the analyses, we adjusted for the complex nature of the survey design by accounting for clustering, stratification, and weighting. Due to the comparisons and combination (pooled data) of surveys from different regions, with different target population sizes, the weights were denormalized. This was done by dividing the women’s standard weights and their total number the country by the respective survey sampling fraction. Data Extraction, recoding, and both descriptive and analytical analysis were carried out using STATA version 14 software. The multilevel analysis was fitted due to the hierarchical nature of the Malaria Indicator Survey data. In this study, the multilevel mixed-effects model was employed and the dependent variable was binary.

The Intraclass Correlation Coefficient (ICC) was employed to assess the variability across the region. Bi variable analysis was first done for maternal age, region, place of residence, educational status, religion, sex of household, wealth status, sources of drinking water and types of toilet facility to select variables for multivariable analysis and variables with p-value less than 0.05 were considered for multivariable analysis.

### Spatial analysis

In Stata 14, the weighted frequency of anemia, cluster number, and geographic coordinate data were combined. Data was then exported to Excel and imported into ArcGIS 10.7 for spatial analysis.

### Spatial autocorrelation analysis

The spatial autocorrelation (Global Moran’s I) statistic examines the distribution of anemia among women of reproductive age in Mali. Moran’s I is a spatial statistic that uses the entire data set to generate a single output value that varies from − 1 to + 1 in order to evaluate spatial autocorrelation. I, Moran’s Values around − 1 suggest scattered anemia, whereas values near + 1 indicate clustered anemia, and values near 0 indicate random distribution of anemia. A statistically significant Moran’s I (p < 0.05) lead to the failure to reject the alternative hypothesis and rejection of the null hypothesis (anemia is randomly distributed) and indicates the presence of spatial autocorrelation.

### Hot spot analysis (Getis-OrdGi* statistic)

The GI* statistics for each area were computed to determine how spatial autocorrelation varies in Mali using Getis-OrdGi* statistics. The p-value is estimated for significance using Z-score in order to determine the statistical significance of clustering. High GI* statistical output suggests a " hot area,“ whereas low GI* statistical output indicates a " cold spot.“

### Spatial interpolation

To determine the impact of a particular event throughout the country, it is highly expensive and time-consuming to gather trustworthy data. As a result, using the observed data, interpolation was utilized to estimate a portion of a certain area. Based on sampled EAs from DHS, the spatial interpolation approach forecasts anemia in the un-studied portions of the country. In this work, anemia in unobserved regions of Mali was predicted using the standard Kriging spatial interpolation approach. The burden of anemia in unsampled regions was estimated for this study using the standard Kriging approach.

### Ethical consideration

The measure of Malaria Indicator Survey program used secondary publically accessible survey data, thus ethical review and participant permission were not required for this particular study. We asked DHS Program for permission to obtain and use the data for this study from their website, and they approved.

## Result

### Sociodemographic characteristics

This study includes a total weighted sample of 10,765 reproductive age women from the 2021 Mali demographic and health survey. 2155 (20.0%) of the total study participants were between the age range of 15–19 years,1959(18.2%) were from Koulikoro region, 8103 (75.3%) were from rural areas, 6941 (64.5%) were not attending formal education, 10,196 (94.7%) were Muslim, 10,099(93.8%) were male headed household, 1960 (18.2%) were poorest, 3567 (33.1%) had unimproved drinking water sources, 5101(47.4%) had unimproved toilets facility, 4094(38.0%) were anemic. Of them, 153(1.4%), were severely anemic, while 2530 (23.5%), and 1410 (13.1%) were moderately and mildly anemic, respectively in Mali (Table 1).

**Table 1:** Sociodemographic characteristics of reproductive age women in Mali 2022. n = 10,765

List of variables		Frequency	Percentage
**Age**	15–19	2155	20.0
	20–24	1979	18.4
	25–29	1961	18.2
	30–34	1628	15.1
	35–39	1307	12.1
	40–44	1053	9.8
	45–49	683	6.3
**Region**	Kayes	1523	14.1
	Koulikoro	1959	18.2
	Sikasso	1795	16.7
	Segou	1847	17.2
	Mopti	1287	12.0
	Tombouctou	716	6.6
	Gao	275	2.6
	Kidal	15	0.1
	Bamako	1348	12.5
**place of residence**	Urban	2662	24.7
	Rural	8103	75.3
**Educational status**	Not educated	6941	64.5
	Primary	1421	13.2
	Secondary	2164	20.1
	Higher	239	2.2
**Religion**	Muslim	10,196	94.7
	Catholic	201	1.9
	Protestant	67	0.6
	Other Christian religion	17	0.2
	Animist	36	0.3
	No religion	249	2.3
**Sex of household head**	Male	10,099	93.8
	Female	666	6.2
**Wealth index**	Poorest	1960	18.2
	Poorer	2042	19.0
	Middle	2105	19.6
	Richer	2217	20.6
	Richest	2441	22.7
**Sources drinking water**	Improved	7198	66.9
	Unimproved	3567	33.1
**Types of toilet facility**	Improved	5664	52.6
	Unimproved	5101	47.4
**Anemia status**	Anemic	4094	38.0
	non-anemic	6671	62.0
**Anemia level**	Severe	153	1.4
	Moderate	2530	23.5
	Mild	1410	13.1
	Not anemic	6671	62.0

### Spatial analysis results

#### Spatial distribution of anemia

**Fig. 1:**
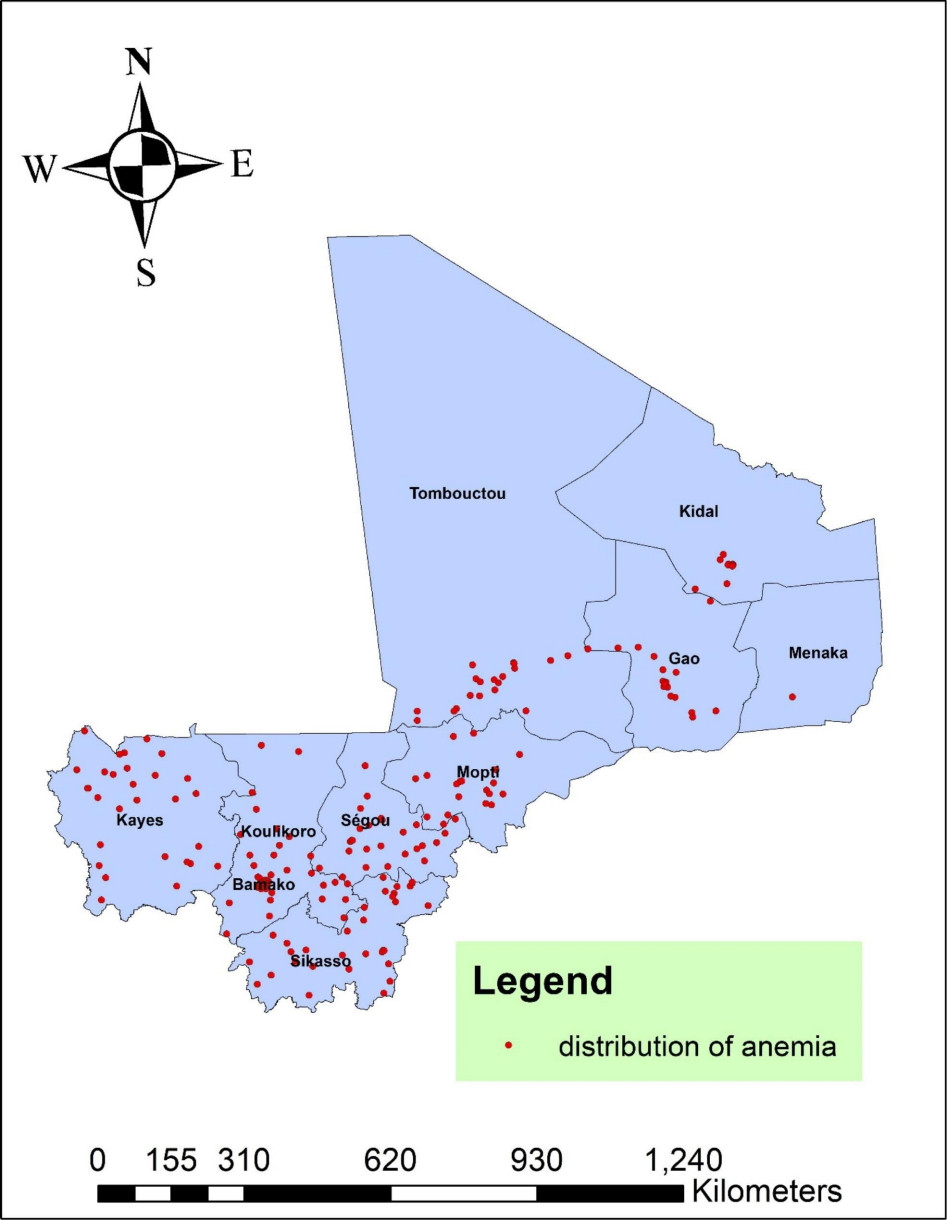
Spatial distribution of anemia among reproductive age women in Mali, 2022

In Mali, anemia status was analyzed geographically using 261 clusters. The number of anemia instances in each cluster corresponds to one enumeration area at each spot on the map. This study’s analysis of the spatial distribution of anemia showed that a higher proportion of anemia in southern and south west region of Mali. The northern and north east region of Mali had a low of proportion of anemia (Fig. 1).

#### Spatial autocorrelation anemia

**Fig. 2:**
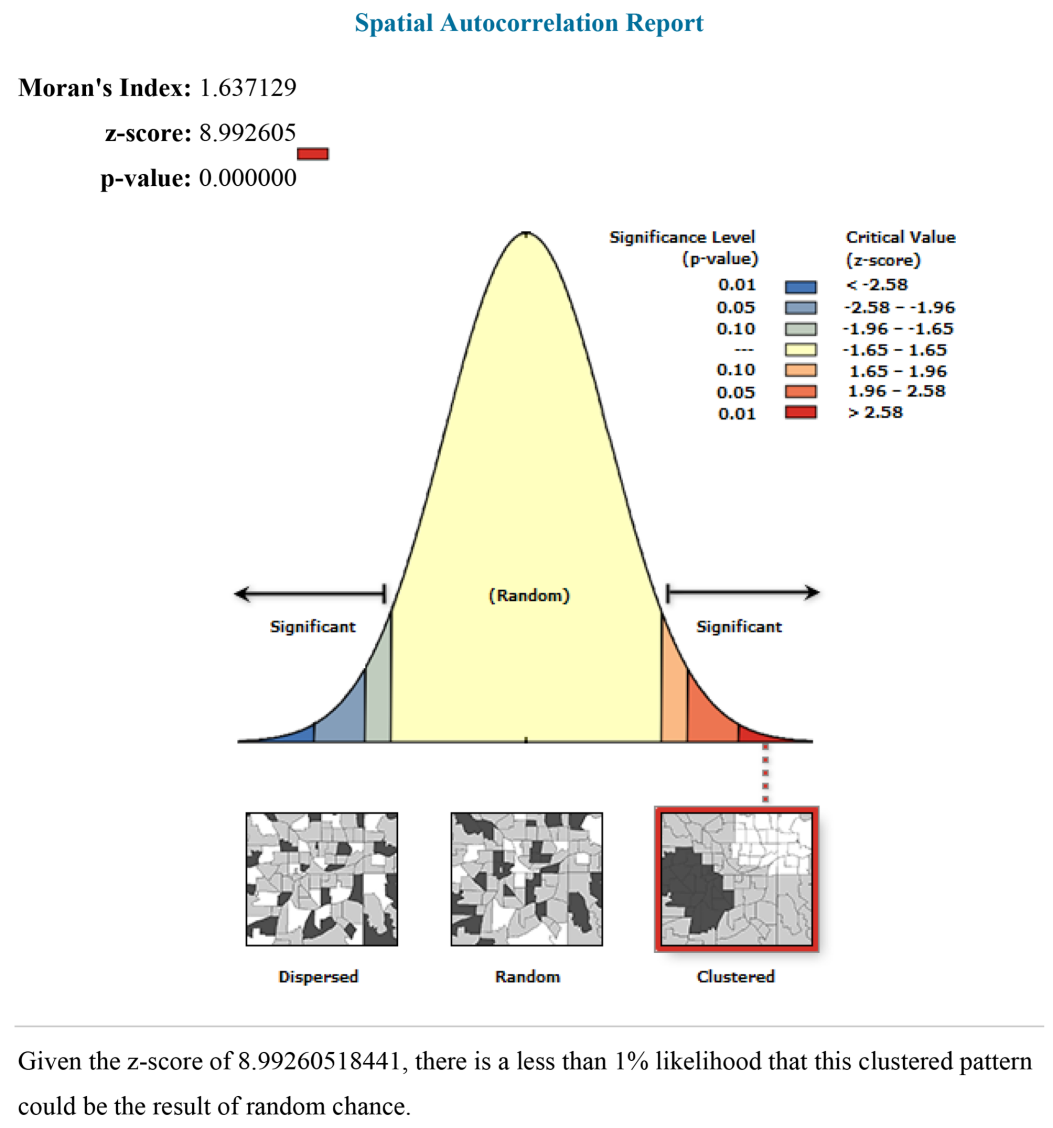
Spatial autocorrelation of anemia in Mali, 2022

The spatial autocorrelation result reveals whether anemia in Mali is randomly distributed across the region, clustered, or dispersed. The results of the spatial autocorrelation study showed a clustering effect in the anemia across the country. The clustered patterns (on the right’s red box side) demonstrated a clustering effect on the anemia in Mali. The outputs have automatically generated keys on the right and left sides of each panel. The probability that this clustered pattern is the result of random chance is less than 1%, according to the z-score of 8.992 (p-value < 0.001). The bright red and blue colors to the end tails indicate an increased level of significances (Fig. 2) .

#### The hotspot analysis result

**Fig. 3:**
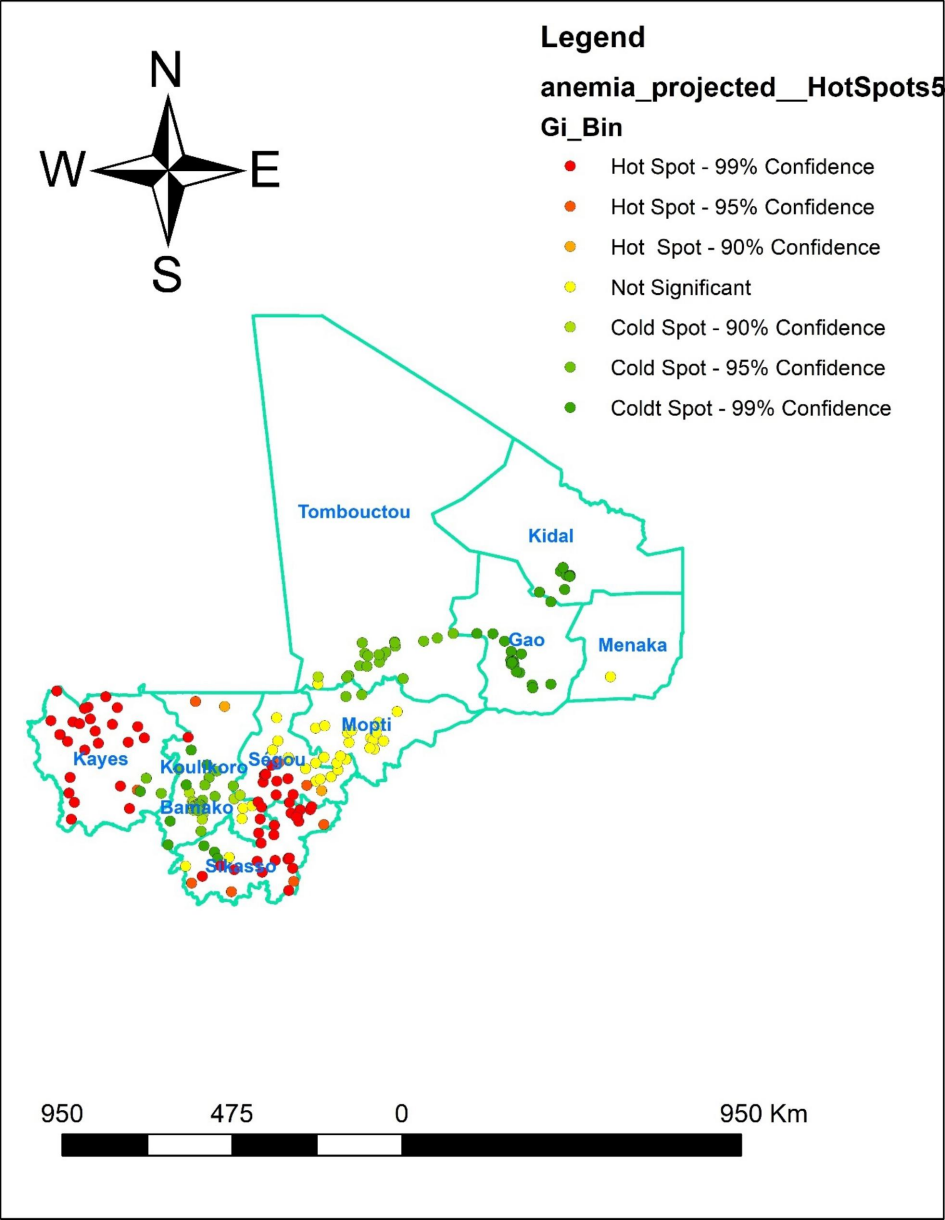
Hotspot analysis of anemia in Mali, 2022

The hotspot analysis result shows the high proportion (hotspot) and low proportion (cold spot) areas of anemia in Mali. The red colors were seen in the Kayes, in few parts of Koulikore, Sikasso and Segou, which are hot spot areas (high proportion of anemia). The green-colored were cold spots (areas with a low percentage of women with anemia) were found in southern part of Tombouctou and in southern part of Koulikore, Bamako, northern part of Sikasso and Gao (Fig. 3).

#### Spatial interpolation or prediction

**Fig. 4:**
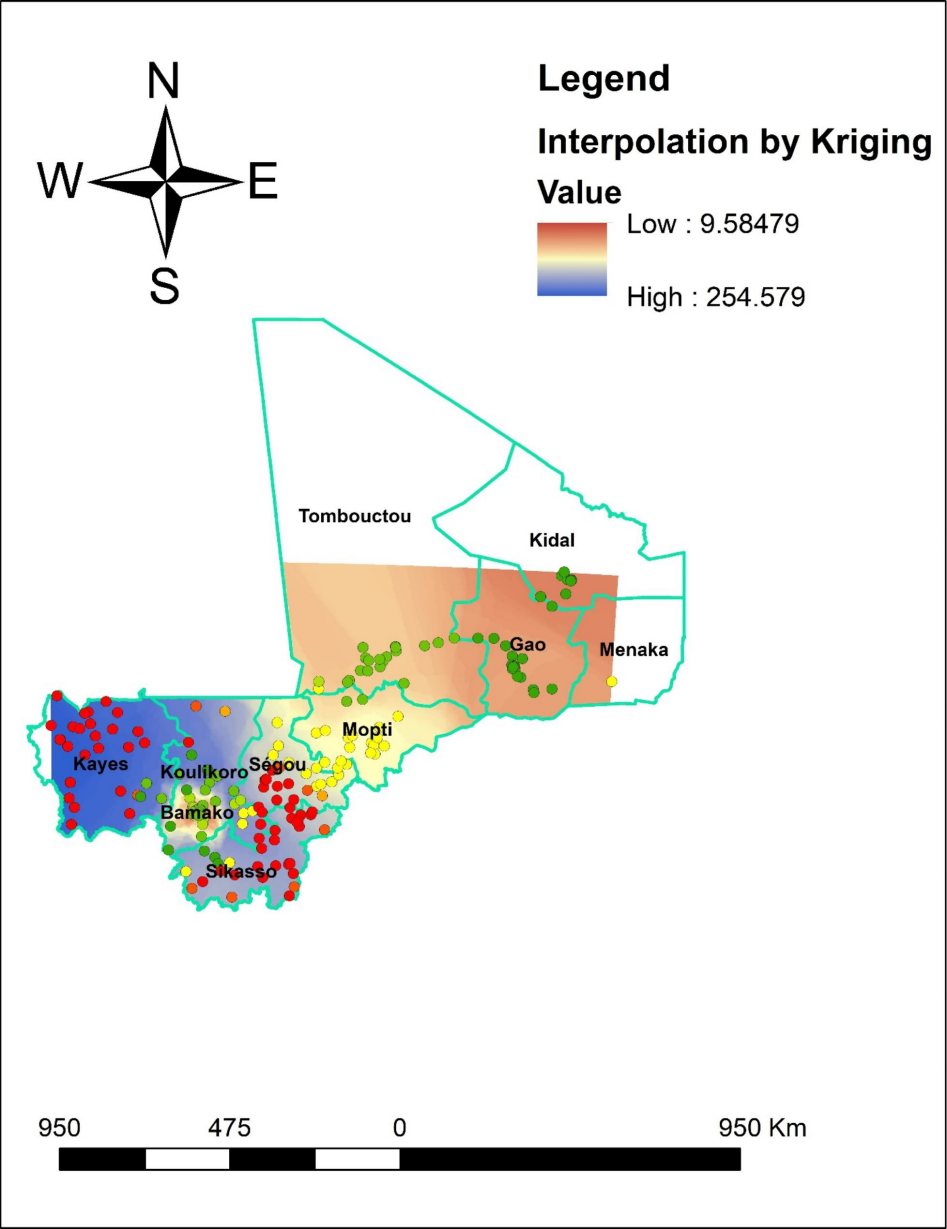
Spatial interpolation of anemia in Mali, 2022

Based on the sampled region, the spatial interpolation approach predicts the proportion of anemia for unsampled areas. The area map was described using the standard Kriging method. The red color represents the projected low prevalence of anemia. If the area’s color shifted from red to blue, it indicates that more people in the area are anemic than was previously expected. The country is predicted to anemia at a low prevalence, as shown by the red color. According to the prediction’s results, southern part Tombouctou, Gao, eastern part of monak, northern Mopti and Bamako have high prevalence of anemia. The blue color prediction showed that the regions of Kayes, Koulikore, Sikasso and Segou had the higher prevalence of anemia nationwide(Fig. 4).

### Model comparison

Four models were built for this multistage investigation. The first model was built. Without independent factors, it is possible to determine how community variation affects women’s anemia status. The second model included variables at the individual level. Community level characteristics were incorporated in the third model. Finally, the fourth model took into account factors at both the individual and community levels. The ICC in the null model showed that among women of reproductive age, there was a variance in anemia status of 7.63% in the communities. The variance in anemia status among women of reproductive age is described by variables at the individual level in 10.62% of occurrences. The difference in anemia status among women of reproductive age is accounted by 9.35% community level variables. In the end, 12.47% of the variances among women in reproductive age were caused by variables at the individual and community levels. Deviance was used to evaluate model fitness for model comparison (AIC). As a result, it was determined that Model IV, which included factors at both the individual and community levels and had the lowest deviance (AIC) value, provided the best suit. Variables having a p < 0.05 significance level were considered to be significant predictors of anemia status among reproductive-age women (Table 2).

**Table 2:** revealed the random effect of anemia and model comparison

Parameters	model I	model II	model III	Model IV
ICC (%)	7.63	10.62	9.35	12.47
Model fitness				
**AIC**	6994.67	58892.35	4055.41	4000.91

### Pearson Chi-Square analysis of factors associated with anemia

The Pearson Chi-square analysis was employed for age, place of residence, region, religion, educational status, sex of household head, wealth index, sources of drinking water and types of toilet facility among reproductive age women. The result of the Pearson Chi-square analysis demonstrated that anemia had significant association with place of residence, region, religion, educational status, sex of household, wealth index, sources of drinking water and types of toilet facility among reproductive age women (Table 3).

**Table 3:** Pearson Chi-Square analysis of factors associated with anemia among reproductive age women in Mali 2022

List of variables	Pearson Chi-Square	DF	P-value
**Age**	1048.697	6	0.000
**Type of place of residence**	120.349	8	0.000
**Region**	120.349	8	0.000
**Religion**	6.684	5	0.031
**Educational attainment**	164.129	3	0.000
**Sex of household head**	60.774	1	0.000
**Wealth index**	210.990	4	0.000
**Sources of drinking water**	44.102	1	0.000
**Types of toilet facility**	39.997	1	0.000

DF = degree of freedom; statistically significance= *p-value < 0.05; **P < 0.01; ***P < 0.001

### Bivariable and multivariable logistic regression

Bivariable logistic regression was employed for age, place of residence, religion, educational status, sex of household, wealth index, sources of drinking water and type of toilet facility among reproductive age women. The result of the bivariable analysis demonstrated that anemia had significant relationships with age, place of residence, region, religion, educational status, sex of household head, wealth index, sources of drinking water and type of toilet facility among reproductive age women. Variables having a p-value less than 0.05 were considered in multivariate analysis. According to the result of the multivariable regression the key variables related with anemia among reproductive age women were women’s age, place of residence, educational status, sex of household head, wealth index, sources of drinking water and type of toilet facility. The odd of anemia among reproductive women who were found between the age range of 20–24 years were 0.8 times less likely [ AOR = 0.817; 95% CI = (0.638,1.047); P = 0.000] relative women who were found between the age range of 15–19 years. The odd of Anemia among reproductive age women who were live in rural area were one times more likely [ AOR = 1.053; 95% CI = (0.880,1.260); P = 0.000] relative to women who were live in the urban area. The odd of anemia among reproductive age women who were attending higher education were 0.4 times less likely to be anemic [AOR = 0.401; 95% CI= (0.278,0.579); P = 0.000] compared to women who were not attending formal education. The odd of anemia among reproductive age women who were Animist were 3.1 times more likely to be anemic [AOR = 3.10; 95% CI= (0.763,12.623) P = 0.04] relative to women who were Muslim. The odd of anemia among male headed household were 0.65 times less likely to be anemic [AOR = 0.653; 95% CI= (0.536,0.794); P = 0.000] compared to female headed household. The odd of anemia among richest were 0.62 times less likely to anemic [ AOR = 0.629; 95% CI= (0.524,0.754) P = 0.000] relative to women who were poorest. The odd of anemia among women who were used unimproved drinking water sources were 1.1 times more likely [AOR = 1.117; CI= (1.017,1.228); P = 0.021} compared to women who used improved drinking water sources. The odd of anemia among women who were used unimproved toilet facility were 1 time more likely [AOR = 1.018; CI= (0.917,1.130); P = 0.041} compared to women who used improved toilet facility (Table 4).

**Table 4:** Bivariable and multivariable analysis of factors associated with Anemia among reproductive age women in Mali 2022. (n = 10,765)

Variables	Categories	Anemia status		COR with 95% CI	AOR with 95% CI
		Yes	No		
Age	15–19	340	1815	1	1
	20–24	819	1160	0.896 (0.702,1.142)	0.817(0.638,1.047) ***
	25–29	1064	897	0.237(0.188,0.299) ***	0.210(0.166,0.267) ***
	30–34	850	778	0.141(0.112,0.178) ***	0.127(0.100,0.161) ***
	35–39	606	701	0.153(0.121,0.194) ***	0.142(0.112,0.181) ***
	40–44	318	735	0.194(0.152,0.246) ***	0.185(0.145,0.236) ***
	45–49	98	585	0.387(0.301, 0.498) ***	0.377(0.293,0.485) ***
Region	Kayes	596	927	1	1
	Koulikoro	810	1149	0.553(0.471,0.648) ***	0.982(0.792,1.219) ***
	Sikasso	737	1058	0.504(0.433,0.586) ***	0.830(0.674,1.023) **
	Segou	775	1072	0.510(0.438,0.595) ***	0.909(0.740,1.116) ***
	Mopti	490	798	0.492(0.422,0.573) ***	0.854(0.694,1.051) *
	Tombouctou	244	472	0.579 (0.491,0.683) ***	1.108(0.887,1.384) *
	Gao	84	191	0.686(0.563,0.834) ***	1.228(0.957,1.575) **
	Kidal	5	10	0.807(0.607,1.071)	1.195(0.866,1.648)
	Bamako	353	994	0.663(0.228,1.930)	1.049(0.333,3.301)
Place of residence	Urban	737	1925	1	
	Rural	3357	4746	1.849(1.680,2.034) ***	1.053(0.880,1.260) **
Educational status	Not educated	1050	2,590	1	1
	Primary	666	2,679	0.277(0.195,0.391) ***	0.331(0.230,0.478) ***
	Secondary	237	912	0.287(0.200,0.411) ***	0.296(0.203,0.431) ***
	Higher	107	643	0.486(0.340,0.694) ***	0.401(0.278,0.579) ***
Religion	Muslim	3878	6318	1	1
	Catholic	69	132	1.198(0.929,1.545)	1.086(0.823,1.434)
	Protestant	24	43	1.406(0.957,2.066)	1.286(0.848,1.952)
	Other Christian religion	3	14	1.300(0.744,2.271)	1.158(0.633,2.117)
	Animist	15	21	3.901(1.024,14.863) **	3.104(0.763,12.623) *
	No religion	106	143	1.032(0.506,2.105)	1.171(0.548,2.506)
Sex of household head	Male	3935	6163	0.490(0.408,0.588) ***	0.653(0.536,0.794) ***
	Female	159	508	1	1
Wealth index	Poorest	798	1163	1	1
	Poorer	861	1181	0.504(0.444,0.573) ***	0.601(0.485,744) ***
	Middle	921	1184	0.474(0.418,0.538) ***	0.585(0.473,0.723) ***
	Richer	886	1331	0.445(0.392,0.504) ***	0.564(0.460,0.692) ***
	Richest	627	1813	0.519(0.459,0.588) ***	0.629(0.524,0.754) ***
sources drinking water	Improved	2580	4618	1	1
	Unimproved	1514	2053	1.433(1.320,1.216) ***	1.117(1.017,1.228) *
Type of toilet facility	Improved	1995	3669	1	1
	Unimproved	2099	3002	1.286(1.190,1.391) ***	1.018(0.917,1.130) *

AOR = Adjusted odd ratio; COR = Crude odd ratio; CI = confidence interval; statistically significance= *p-value < 0.05; **P < 0.01; ***P < 0.001

## Discussion

The study assessed the spatial distribution of anemia and the factors that influence its among women of reproductive age in Mali, evidenced by Mali Malaria Indicator Survey 2021. This survey revealed that 38.0% of women of reproductive age were anemic. Of them, 153(1.4%), were severely anemic, while 2530 (23.5%), and 1410 (13.1%) were moderately and mildly anemic, respectively. This finding was in line with which was conducted in Jordan [16]. This finding was higher than the studies which was conducted in Ethiopia [17], Rwanda [18], Uganda [8] and in Brazil [19]. On the other hand the finding of this study was lower than the study which was conducted in in Bangladesh [20] and in India [21]. These discrepancies may have been brought on by the study time, sample size, geographical, cultural, and dietary differences between countries [22]. in addition to this, anemia is also brought on by the inability to obtain iron-rich foods due to their low socioeconomic status [23], the inadequate to access and utilization of health care, and the high prevalence of infectious disease.

According to this study, reproductive women between the ages of 20 and 24 were less likely to have anemia than those between the ages of 15 and 19. This finding was supported by the study which was conducted in conducted in India [24]. Women younger than 18 years had received less ANC visits [25]. This results in young women not receiving iron supplements during their pregnancies, women not receiving nutritional counseling regarding food variety, and an increase in home deliveries due to a lack of ANC follow-up, which causes post-partum hemorrhages, all of which increase the risk of anemia in younger women.

This study revealed that Women of reproductive age who lived in rural areas had a higher likelihood of anemia than those who lived in urban areas. This finding was concurrent with study which was conducted in Lao People’s Democratic Republic [26], in Ethiopia [27], in Bangladesh [28], Low socioeconomic position, lack of access to hygienic sanitation facilities, and a lack of nutrient-rich food choices are all associated with higher disease rates, which may also raise the risk of anemia in rural areas [29]. More women are forced to work in the fields in rural areas, where poverty is also more prevalent. Between fields and homes, there is a great deal of space. Without using a vehicle, they must travel there. They carry heavy weights as they go toward the fields [30]. in addition to this low access to mass media, and nutritional information were the potential factors to increase the risk of anemia among reproductive age women. In contrast to this finding the study which was conducted in Malawi demonstrated that women from rural areas had lower odds of anemia as compared with those who were from urban areas [31].

This study showed that women of reproductive age who were engaged in higher education were less likely to be anemic than women who were not attending formal education. This finding was consistent with study which was conducted in Tanzania [32], and in Ethiopia [33]. The first potential explanation for this variation was that women with higher levels of maternal education would be more knowledgeable about the diversity of foods that are high in folic acid and iron. Additionally, acquiring higher education may assist women in forming healthy lifestyle habits, such as better hygiene and health-seeking behaviors, which can protect them from developing anemia.

According to this study, women of reproductive age who were Animist were more likely to be anemic than those who were Muslim. This conclusion may not be directly related to religion, but it is likely influenced by various food habits, food taboos, and other factors.

The findings of this research indicated that male-headed households had a lower likelihood of anemia than female-headed households. This finding was concurrent with study which was conducted in east Africa [34], in Ethiopia [35] and in Nepal [36]. This might be that Male-headed households had higher awareness on anemia and treatment-seeking behaviors for any health problems than female-headed households [37].

This research showed that wealthy women had a reduced risk of anemia than did women from the poorest socioeconomic groups. This finding was consistent with the study which was conducted in Tanzania [38], in Ghana [39], and in India [40]. This might be the household’s ability to access things vital to the health and wellbeing of its members, such food and healthcare, depends on its level of wealth [41]. In addition, Women with high socioeconomic status could afford a variety of foods, both in terms of quality and quantity. Evidence also suggests that low-income households are less likely to seek and timely their hospital treatment, which could prevent them from receiving treatment for conditions that might result in anemia [42].Furthermore, Poor women are more likely to face several challenges in terms of their decision-making autonomy in the home and the responsibility of providing for the fundamental requirements of household members falls unfairly on women that increase the risk of anemia.

This study found that there was a greater likelihood of anemia in women who used unimproved drinking water sources and unimproved toilets facility as compared to women who used improved drinking water sources and improved toilets facility. This finding was supported by the study which was conducted in Rwanda [43], in Ethiopia [27] and in Uganda [8]. This may be due to the increased risk of both foodborne and waterborne illnesses in women who lack improved access to toilet facilities and safe drinking water sources, which may also contribute to anemia. Additionally, these groups of women are susceptible to helminthic diseases like hookworm, which is the most prevalent cause of anemia in unsanitary conditions.

### Strengths and limitations of this study

- The Mali Malaria Indicator Survey has a similar design, with identical variables in a different environment; the result may, therefore, be applicable to other similar locations.
- The study used a sufficiently large sample size at the national level to ensure its representativeness.
- Recall bias is one of the potential drawbacks, especially for retrospective data based on past experiences.
- The magnitude of the bias is often unknown and correcting for the bias is difficult.
- Since, this study was cross sectional study.it doesn’t showed temporal relationships between independent and dependent variable.

## Conclusion

In this study sociodemographic factors were associated with anemia and also there were spatial variations in prevalence of anemia across the region among reproductive-age women. Empowering women to have better educational status, improving the wealth index, creating awareness among rural residences women about improved drinking water sources and improved toilet facility, promoting education about anemia through religiously acceptable persons, and employing an integrated approach in prevention and intervention to reduce anemia in high prevalent region of Mali were the key factors to reduce the burden of anemia and related maternal and perinatal mortality associated with anemia.

## Data Availability

The data were obtained from Mali Malaria Indicator Survey 2021 that was found at DHS portal of (https://dhsprogram.com/data/dataset_admin/index.cfm).
